# Unique Intravascular Ultrasound and Optical Coherence Tomography Features of Multivessel Coronary Stenosis in Pseudoxanthoma Elasticum

**DOI:** 10.1016/j.jaccas.2025.106231

**Published:** 2025-11-28

**Authors:** Keita Tashiro, Ryutaro Ikegami, Makoto Hoyano, Hideki Usuda, Yuzo Washiyama, Takumi Akiyama, Kota Nishida, Naoki Kubota, Takeshi Okubo, Takayuki Inomata

**Affiliations:** Department of Cardiovascular Medicine, Niigata University Graduate School of Medical and Dental Sciences, Niigata, Japan

**Keywords:** atherosclerosis, imaging, intravascular ultrasound, percutaneous coronary intervention, stenosis

## Abstract

**Background:**

Pseudoxanthoma elasticum (PXE) is a rare disease resulting from *ABCC6* gene mutations; it causes elastic fiber calcification with unclear arterial stenosis mechanisms.

**Case Summary:**

A 60-year-old man with PXE developed multivessel coronary stenosis despite low atherosclerotic risk. Coronary computed tomography demonstrated noncalcified stenosis in all vessels, intravascular ultrasound (IVUS) showed concentric high-intensity lesions with mixed acoustic shadowing, and optical coherence tomography (OCT) indicated thin, sheet-like calcifications with low-intensity heterogeneous tissue. Guidance through IVUS/OCT imaging contributed to successful percutaneous coronary intervention.

**Discussion:**

PXE demonstrates stenotic mechanisms distinct from conventional atherosclerosis. Stenosis develops as a result of tissue thickening caused by calcification-induced fragmentation and repair. To our knowledge, no previous reports have evaluated coronary stenosis in PXE using both IVUS and OCT.

**Take-Home Messages:**

Patients with PXE may develop significant coronary stenosis despite low atherosclerotic risk, requiring early cardiovascular evaluation. Combined IVUS/OCT imaging reveal unique stenotic findings resulting from elastic fiber calcification.

## Visual Summary

**Figure:**
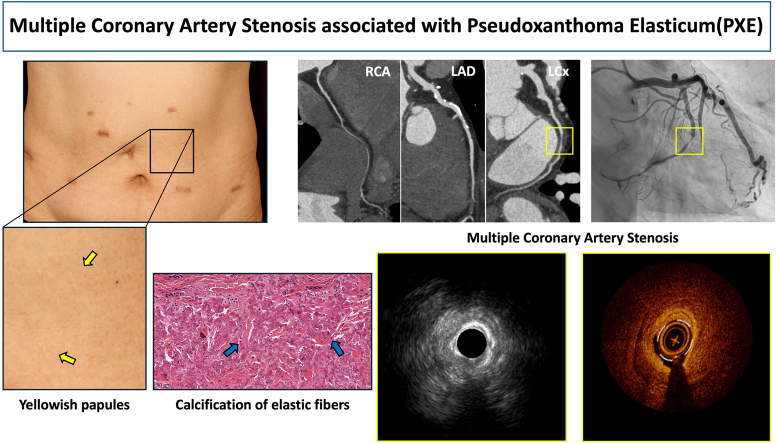

## History of Presentation

**Figure 1:**
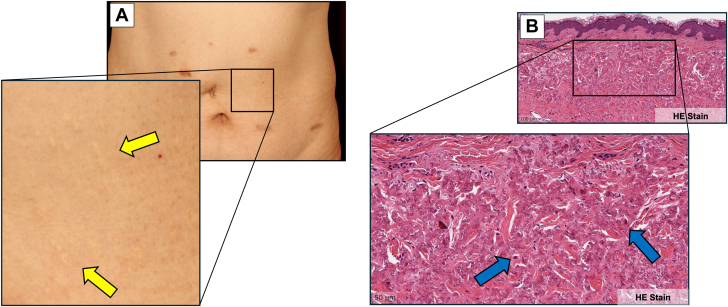
Initial Cutaneous and Skin Biopsy Findings (A) Macroscopic cutaneous findings. Characteristic scattered yellowish papules (yellow arrows) emerged throughout the body. (B) Pathological observation of skin biopsy from the same region revealed elastic fiber thickening throughout the media, with scattered calcifications (blue arrows) on hematoxylin and eosin (HE) staining.

Old cerebral infarction and chronic ischemic changes on brain magnetic resonance imaging performed 5 years earlier were incidentally found in a 60-year-old Asian man. He remained asymptomatic and was managed conservatively. Approximately 1 year ago, scattered yellowish papules emerged throughout his body (Figure 1A). Skin biopsy revealed that medial elastic fibers had thickened and calcified (Figure 1B). He had been evaluated at a local ophthalmology clinic for mild visual impairment, and fundoscopic examination revealed angioid streaks. The patient was referred to our department for cardiovascular evaluation. He presented with normal vital signs and no cardiac murmurs, although bilateral radial artery pulsations were weak.

## Past Medical History

The patient's medical history included old cerebral infarction, hypertension, and vertigo but no family history of pseudoxanthoma elasticum (PXE) or cardiovascular disease. Although he had a history of mild smoking, he had been abstinent for an extended period.

## Differential Diagnosis

The combined findings of scattered yellowish papules throughout the body, angioid streaks (as an ocular complication), and old cerebral infarction (as a cardiovascular complication) strongly suggested PXE. Differential diagnoses included sickle cell disease, thalassemia, Ehlers-Danlos syndrome, Paget disease, and drug-induced conditions such as D-penicillamine. However, on the basis of the characteristic skin biopsy findings, he was ultimately diagnosed with PXE both clinically and pathologically. For coexisting cardiovascular lesions, additional evaluation was performed.

## Investigations

**Figure 2:**
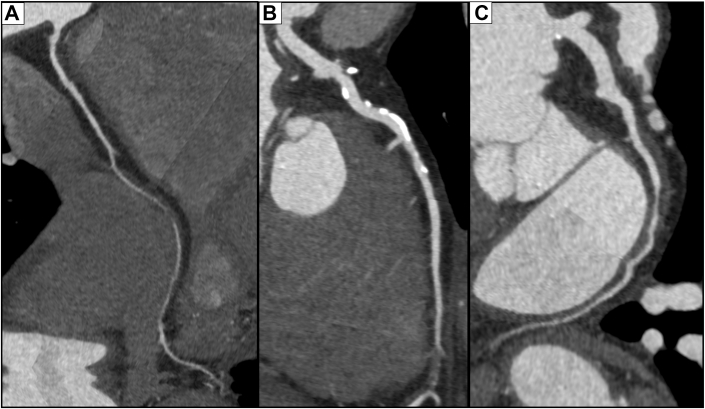
Findings on Coronary Computed Tomography Coronary CT showed noncalcified stenosis in the (A) RCA, (B) LAD, and (C) LCx, with intimal calcifications in the proximal LAD. CT = computed tomography; LAD = left anterior descending artery; LCx = left circumflex artery; RCA = right coronary artery.

Laboratory tests revealed no significant atherosclerotic risk factors (blood urea nitrogen: 16 mg/dL, creatinine: 0.61 mg/dL, glycated hemoglobin: 6.1%, glucose: 98 mg/dL, high-density lipoprotein cholesterol: 61 mg/dL, and low-density lipoprotein cholesterol: 90 mg/dL). Although the patient was asymptomatic, coronary computed tomography (CT) revealed scattered intimal calcification and multiple stenotic or occlusive lesions without apparent calcification in the right coronary artery, left anterior descending artery, and left circumflex artery (LCx) (Figures 2A to 2C). Coronary angiography detected severe stenosis or occlusive lesions in all 3 vessels, accompanied by well-developed collateral circulation (Video 1, Video 2, Video 3); however, collateral circulation to the LCx was insufficient. Moreover, echocardiography revealed mild wall motion abnormality in the posterior wall, and coronary CT revealed nontransmural delayed enhancement in the same area—findings consistent with an old myocardial infarction with preserved viability. Both whole-body CT and angiography revealed calcification in the descending aorta without stenosis; meanwhile, the major branches and bilateral internal mammary arteries showed no calcification or stenosis.

## Management

Given the preserved myocardial viability and insufficient collateral circulation development to this territory, we decided to perform percutaneous coronary intervention (PCI) on the LCx. In contrast, although the left anterior descending artery showed chronic total occlusion in the distal segment and the right coronary artery was diffusely small-caliber with severe stenosis in the distal portion, we decided to manage these vessels with optimal medical therapy. We made this decision considering that the patient was asymptomatic, the left ventricular ejection fraction was globally preserved, and the lesions were distal with well-developed collateral circulation.

Both intravascular ultrasound (IVUS) and optical coherence tomography (OCT) were used to evaluate the LCx during the procedure. IVUS revealed scattered high-intensity areas in the intima/media of nonculprit areas (Video 2). The culprit lesion appeared as a concentric high-intensity lesion, containing both shadowed and nonshadowed regions on imaging. Meanwhile, OCT showed sheet-like calcifications corresponding to IVUS high-intensity areas in nonculprit areas (Video 3). The culprit lesion exhibited thin sheet-like calcifications on the intimal side, with low-intensity, minimally attenuating, heterogeneous tissue in the outer regions. After predilatation with a scoring balloon, we applied a drug-coated balloon. Consequently, the stenosis improved, and the procedure was completed successfully.

## Outcome and Follow-Up

No anginal or heart failure symptoms have been observed after the procedure. The patient is being carefully followed up while receiving standard secondary prevention therapy, similar to conventional ischemic heart disease. As a specific therapy for PXE, etidronate administration is being considered.^1^

## Discussion

In PXE, calcification occurs directly within elastic tissues because of decreased pyrophosphate levels, significantly different from the mechanism of calcification in conventional atherosclerosis.^2^ Therefore, PXE can cause coronary stenosis even in young patients without atherosclerotic risk factors.^3^ This condition reportedly occurred in a relatively young patient without atherosclerotic risk factors other than hypertension. The indication for revascularization in PXE-related coronary artery disease should be determined in the same manner as conventional coronary artery disease. In chronic coronary syndrome, comprehensive assessment including symptoms, evidence of myocardial ischemia and viability, and cardiac function is desirable. In our case, all 3 vessels (right coronary artery, left anterior descending artery, and LCx) showed occlusive or severe stenotic lesions with complex collateral circulation development. Therefore, without performing functional ischemia evaluation, we concluded that revascularization of the circumflex territory would provide the greatest benefit owing to relatively greater ischemia in this region, and proceeded with PCI to this site.

The primary reason for our use of a drug-coated balloon instead of a drug-eluting stent was the small vessel diameter (approximately 2 mm) in the distal lesion. Additionally, vascular fragility associated with PXE increases the risk of gastrointestinal and urinary bleeding^2^; thus, the duration of dual antiplatelet therapy during PCI becomes a critical factor. Given the characteristics of PXE, lesions are unlikely to pose high thrombotic risk as with typical plaque rupture-induced occlusions, and short-term dual antiplatelet therapy may be preferable. In our case, considering these bleeding concerns and the relatively small vessel diameter, we chose to complete the procedure with a drug-coated balloon rather than stent implantation.

Arterial calcification ranges from microscopic to large deposits, with differences in detection capabilities between imaging modalities. Histological calcification can develop even in areas where CT indicates no apparent calcification, demonstrating the limitations of CT detection sensitivity.^4^ Although CT clearly shows that patients with PXE have more arterial calcifications than those without,^5^ calcification may occur throughout systemic tissues beyond what CT can detect.

Intravascular coronary imaging in patients with PXE remains rarely reported. Previous IVUS studies of stenotic segments have described high-intensity lesions containing a combination of areas with and without acoustic shadowing.^6^^,^^7^ Although these patterns may reflect fragmented sheet-like calcifications and microcalcifications, their precise pathological nature remains unclear. In the present case, the lesions underwent detailed qualitative assessment using OCT in addition to IVUS.

Consistent with previous reports, IVUS demonstrated a concentric high-intensity lesion with a mixture of shadowed and nonshadowed areas. On OCT, the stenotic segment displayed thin sheet-like calcifications along the intimal layer, while the outer layers exhibited low-intensity, minimally attenuating, heterogeneous tissue. In conventional atherosclerotic lesions, low-intensity areas on OCT suggest lipid-rich plaques or calcified nodules. However, the former would also appear low in intensity on IVUS, and the latter would have distinct borders, both inconsistent with the current case. Although microcalcification detection is limited by spatial resolution, OCT can identify deposits as small as 10 to 20 μm, likely appearing as indistinct low-intensity areas.^8^ Autopsy studies of patients with PXE have revealed thickened fibrous tissue with intermixed fine calcifications, suggesting that arterial stenosis is unique in PXE.^9^ Therefore, the IVUS and OCT findings in the present case represent stenotic lesions composed of thickened fibrous tissue containing microcalcifications, characteristic of PXE.^10^ Interestingly, apparently normal vessel walls without atherosclerotic plaques reportedly exhibit fragmented sheet-like calcifications.

These findings support the concept that the calcification mechanism in PXE—ectopic calcification caused by calcium deposition in elastic fibers—differs from that of conventional atherosclerosis. When arterial stenosis develops, it may not primarily result from direct luminal narrowing by calcified nodules, but rather from microcalcification-induced elastic fiber thickening and inflammation. Microcalcifications within elastic fibers may compromise their flexibility and prevent the arterial wall from accommodating physiological stresses. When exposed to repetitive arterial pulsation, such compromised elastic fibers might tear microscopically, potentially triggering localized inflammatory responses and subsequent tissue repair processes. With repetitive cycles of injury and repair over time, the vessel wall progressively thickens.

In our case, coronary intervention was performed in a patient with an established PXE diagnosis. However, PXE may remain unrecognized in clinical practice. Therefore, when coronary artery disease appears disproportionately severe relative to traditional atherosclerotic risk factors, when calcification develops within nonatherosclerotic plaques, or when IVUS and OCT reveal morphologies inconsistent with typical lipid-rich or fibrous plaques, a systemic evaluation for PXE should be considered.

## Conclusions

Combined IVUS/OCT analysis of coronary artery stenosis in a patient with PXE demonstrated characteristic imaging findings, providing important insights for understanding vascular stenotic mechanisms in PXE.

**Visual Summary:**
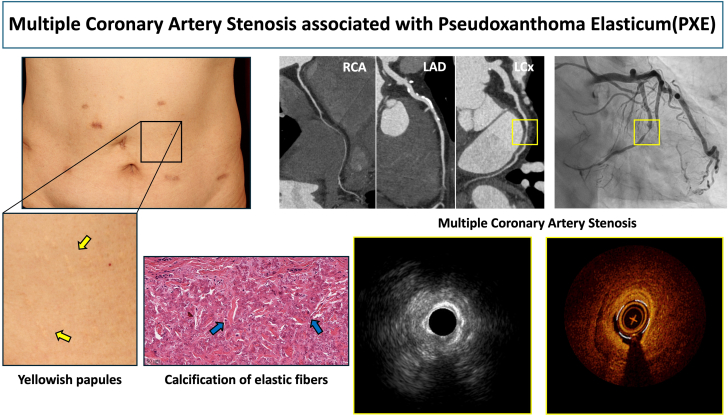
Multiple Coronary Artery Stenosis Associated With Pseudoxanthoma Elasticum LAD = left anterior descending artery; LCx = left circumflex artery; RCA = right coronary artery.

## Funding Support and Author Disclosures

The authors have reported that they have no relationships relevant to the contents of this paper to disclose.

**Take-Home Messages**

• Patients with PXE may develop significant coronary stenosis despite having low conventional atherosclerotic risk factors, making early cardiovascular evaluation important.
• Combined IVUS/OCT imaging reveals unique stenotic mechanisms in PXE distinct from those in conventional atherosclerosis.

## Appendix

Video 1 Coronary Angiography Revealing Multiple Stenotic Lesions in the RCA, LAD, and LCx, With Collateral Circulation Development (Left) RAO 30° view of the RCA, (Middle) LAO 30°/CRA 30° view of the LCA, and (Right) RAO 30°/CAU 30° view of the LCA. CAU = caudal; CRA = cranial; LAO = left anterior oblique; LCA = left coronary artery; RAO = right anterior oblique; RCA = right coronary artery.

Video 2 IVUS Imaging of the LCx From Distal to Proximal IVUS reveals scattered high-intensity areas in nonculprit areas. The culprit lesion appears as a concentric high-intensity lesion, with scattered high-intensity tissue in the outer regions. IVUS = intravascular ultrasound; LCx = left circumflex artery.

Video 3 OCT Imaging of the LCx From Proximal to Distal OCT reveals sheet-like calcifications corresponding to IVUS high-intensity areas in nonculprit areas. The culprit lesion exhibits thin sheet-like calcifications on the intimal side, with low-intensity, minimally attenuating, heterogeneous tissue in the outer regions. LCx = left circumflex artery; OCT = optical coherence tomography.
